# The impact of N-glycosylation on the properties of the antimicrobial peptide LL-III

**DOI:** 10.1038/s41598-023-29984-0

**Published:** 2023-03-06

**Authors:** Attila Tortorella, Linda Leone, Angelina Lombardi, Elio Pizzo, Andrea Bosso, Roland Winter, Luigi Petraccone, Pompea Del Vecchio, Rosario Oliva

**Affiliations:** 1 Department of Chemical Sciences, University of Naples Federico II, Via Cintia 26, 80126 Naples, Italy; 2 Department of Biology, University of Naples Federico II, Via Cintia 26, 80126 Naples, Italy; 3 Centro Servizi Metrologici E Tecnologici Avanzati (CeSMA), University of Naples Federico II, 80126 Naples, Italy; 4 Faculty of Chemistry and Chemical Biology, Biophysical Chemistry, TU Dortmund University, Otto-Hahn-Str. 4a, 44227 Dortmund, Germany; 5 Present Address: Scuola Superiore Meridionale (SSM), Largo San Marcellino 10, 80138 Naples, Italy

**Keywords:** Biophysics, Chemical biology, Chemistry

## Abstract

The misuse of antibiotics has led to the emergence of drug-resistant pathogens. Antimicrobial peptides (AMPs) may represent valuable alternative to antibiotics; nevertheless, the easy degradation due to environmental stress and proteolytic enzyme action, limits their use. So far, different strategies have been developed to overcome this drawback. Among them, glycosylation of AMPs represents a promising approach. In this work, we synthesized and characterized the N-glycosilated form of the antimicrobial peptide LL-III (g-LL-III). The N-acetylglucosamine (NAG) was covalently linked to the Asn residue and the interaction of g-LL-III with bacterial model membranes, together with its resistance to proteases, were investigated. Glycosylation did not affect the peptide mechanism of action and its biological activity against both bacteria and eukaryotic cells. Interestingly, a higher resistance to the activity of proteolytic enzymes was achieved. The reported results pave the way for the successful application of AMPs in medicine and biotechnological fields.

## Introduction

The increasing spread of microorganisms, such as bacteria, resistant to conventional antibiotics has become a critical problem worldwide^1,2^. Indeed, bacteria have developed different mechanisms that can render antibiotics ineffective. Enzymatic modifications of antibiotics, such as acetylation and phosphorylation, is a common strategy used by bacteria which can hamper or alter the interaction of the drug with its target^3,4^. Another strategy is represented by enzymatic degradation, such in the case of β-lactam antibiotics (e.g., penicillin) operated by β-lactamases^5^. Moreover, changes in lipid composition of membranes and mutations of membrane-proteins can affect the permeability and efflux activity of the drugs^3^. Finally, mutations/changes in the targeted macromolecules alter the antibiotic binding capability rendering them ineffective^5^. Thus, the current efforts are devoted to finding new and alternative antimicrobial agents, such as antimicrobial peptides (AMPs). AMPs are a heterogeneous class of small peptides, active against different pathogenic organisms, such as bacteria, viruses, fungi and even against cancer cells^6,7^. AMPs are part of the defense system of all forms of life^7^ and constitute the first set of defense weapons to fight the invasion of foreign pathogens. They are composed of 10–50 (or more) natural L-amino acids, but natural peptides containing also D-amino acids were recently identified^8^. Both hydrophobic and positively charged residues are present in their primary sequence, usually in the ratio 2:1 and 1:1^9^. AMPs are involved in a wide variety of functions (*e.g.,* wound healing, epithelial cell proliferation)^10^. However, they are best known for their antimicrobial activity. This function is carried out mainly by targeting the lipid matrix of the cytoplasmic bacterial membrane^11^, which is enriched in anionic lipids, (*e.g.,* phosphatidylglycerols and phosphatidic acids). In fact, AMPs can selectively interact with bacterial membranes leading to their permeabilization and/or destabilization in a non-specific way (*i.e.*, without a receptor), which decrease the probability to develop resistance mechanisms in bacteria^12,13^. Three different models have been proposed that can describe the action mode of AMPs: the barrel-stave, toroidal and the carpet mechanism^14^. However, a given peptide may act differently, strictly depending on its physico-chemical properties (*e.g.,* charge, hydrophobicity), and the properties of the lipid bilayer (*e.g.*, the lipid’s lateral organization, fluidity and charge density). Thus, a peptide could not act exclusively in the framework of one of the mechanisms reported above. In addition, some peptides have intracellular targets, such as proteins and nucleic acids involved in fundamental pathways of cellular life^15^.

The AMP Lasioglossin-III (LL-III) is a 15-residue peptide found in the venom of the eusocial bee *Lasioglossum laticeps.* It showed a strong activity against both *Gram*-positive and *Gram*-negative bacteria coupled with low hemolytic activity against rat erythrocytes^16^. Remarkably, LL-III shows also antifungal and antitumor activities^16–18^. Recently, it was also reported that LL-III affects the structure and morphology of in vitro membraneless organelles, such as those formed by mixing the protein LAF-1 and RNA^19^, and on the fibrillation pathway of amyloidogenic peptides, such as α-synuclein^20^, opening new avenues for the use of short AMPs in the treatment of neurodegenerative diseases. We previously characterized the mechanism of LL-III with bacterial model membranes^21^ and found that it interacts with bacterial model membranes, adopting a helical conformation, and inducing the formation of lipid domains. Through the destabilization of the membrane, the peptide can access inside the cell, thereby affecting its functionality and ultimately leading to the pathogens’ death.

Even though AMPs are good candidates as alternatives to conventional antibiotics, their application is not straightforward due to their unfavorable pharmacological profile^22,23^. The AMPs stability strongly depends on environmental factors, such as pH, temperature and exposure to UV-radiations causing residues oxidation (*e.g.*, Trp). More importantly, they are prone to hydrolysis catalyzed by proteases present in the human body or secreted by the pathogens that can seriously compromise their activity^23^. To deal with the problem of protease-induced degradation, several strategies have been developed, such as the introduction of unnatural amino acids, β-amino acids or D-enantiomers, as well as protection of both N– and C– termini (*e.g.*, carboxylation and amidation, respectively)^24^. Though attractive, this strategy may lead to highly stable peptides, with consequent undesirable increase of cytotoxicity^25^. Attachment of fatty acids (lipopeptides) or PEG (polyethylene glycol)^26–28^, as well as encapsulation in nanoparticles, hydrogels and cyclodextrins^29^ also represent useful strategies for enhancing AMPs stability. A further promising approach is the glycosylation of the peptide at key residues (*e.g.*, Asn, Ser). Glycosylation is a post-translational protein modification commonly encountered in Nature^30^. Four different kinds of glycosylation are known: N-glycosylation of the Asn residue, O-glycosylation, involving Ser, Tyr and Thr residues, S-glycosylation of free Cys and C-glycosylation of Trp^22,30^. A covalently linked sugar moiety can modulate the peptide properties (such as their hydrophilicity, bioavailability, and membrane permeability) depending on the type of the attached sugar and on the position. The presence of a carbohydrate is effective in enhancing the stability against the proteases action^22,30^, since it can hamper the binding of the enzymes to the targeted peptide residues, rendering them less prone to hydrolysis^31–33^.

In the attempt to increase the stability of LL-III, against protease degradation, we report: (i) the synthesis of the LL-III glycosylated form (g-LL-III) where a N-acetylglucosamine (NAG) was covalently attached to the Asn residue at the N-terminal region of the peptide (peptide sequence: V**N**WKKILGKIIKVVK-NH_2_), (ii) the biophysical characterization of the interaction between g-LL-III and POPC/POPG lipid vesicles serving as bacterial model membranes, (iii) the stability of g-LL-III against two proteolytic enzymes, α-chymotrypsin and pepsin, tested in vitro; iv) ex vivo assays to evaluate the ability of g-LL-III to affect bacteria viability, and to test the in serum stability of the g-LL-III compared to the parent peptide. Overall, our results demonstrated that the proposed chemical modification strategy successfully confers an increased resistance against proteases without altering the antimicrobial activity or the AMPs’ mechanism of action.

## Materials and methods

### Materials

Lasioglossin III (VNWKKILGKIIKVVK-NH_2_) peptide and its glycosylated form g-LL-III were chemically synthesized by solid phase peptide synthesis (SPPS) and purified by reverse phase HPLC (RP-HPLC). All the Fmoc-protected amino acids were purchased from Novabiochem (Merck). The asparagine residue functionalized with N-γ-(2-acetamido-2-deoxy-3,4,6-tri-O-acetyl-beta-D-glucopyranosyl) was purchased from IRIS Biotech. Piperidine, N,N-diisorpyplethylamine (DIEA) and triisopropylsilane (TIS) were purchased from Sigma-Aldrich, as well as the resin and the carboxyl group activators. Trifluoroacetic acid (TFA) and all the solvents used in the synthesis and purification were supplied by Romil. The coupling reagents HATU and HOBt were purchased from Anaspec. Dimethyl formamide (DMF) and N-methyl pyrrolidone (NMP) were used as chromatographic grade solvents and were purchased from Romil.

The lipids 1-palmytoil-2-oleyl-*sn*-glycero-3-phosphocholine (POPC), 1-palmytoil-2-oleyl-*sn*-glycero-3-*rac*-phosphoglycerol (POPG) and 1,2-dipalmytoil-*sn*-glycero-3-phospocholine (DPPC) were purchased from Avanti Polar Lipids Inc. (Alabaster, AL, USA) and were used without further purification. Chloroform, methanol, ethanol and dimethyl sulfoxide (DMSO) were purchased from Sigma Aldrich Chemical. The fluorescent probe Laurdan (6-dodecanoyl-N,N-dimethyl-2-naphtylamine) and the fluorophore 1, 4-bis(5-phenyloxazole-2-yl)benzene (DM-POPOP) for lifetime experiments were purchased from Sigma Aldrich. The biophysical experiments were performed in a 10 mM phosphate buffer solution at pH 7.4.

### Peptide synthesis

LL-III and g-LL-III peptides were synthesized using an ABI 433A peptide synthesizer (Applied Biosystem, Foster City, CA, USA) with standard Fmoc chemistry on a 0.1 mmol scale. The acid labile H-PAL ChemMatrix resin, with a substitution of 0.20 mmol/g, was used as solid support. Amino acids were activated in situ with 2-(7-Aza-1H-benzotriazole-1-yl)-1,1,3,3-tetramethyluronium hexafluorophosphate (HATU) as coupling reagent. Peptide cleavage from the resin and sidechains deprotection was accomplished using a mixture of 94% trifluoroacetic acid (TFA), 2.5% H_2_O, 2.5% triisopropylsilane (TIS), and 1% ethanedithiol (EDT), yielding the peptides with amidated C-terminal. The crude peptides were precipitated in cold diethyl ether and dried under reduced pressure. The synthesis yields were 58% and 63% for LL-III and g-LL-III, respectively, based on HPLC analysis of the crude peptides (Figure. S1 and S2 panel A). Removal of the acetyl groups from protected hydroxyls of N-acetylglucosamine (GlcNAc) was achieved by treating the crude g-LL-III peptide (1.5 mM) with a solution of sodium methoxide (~ 10 mM) in methanol at pH 9. Milder conditions with respect to literature procedures were used to prevent amino acids racemization. The reaction was carried out overnight at room temperature, under magnetic stirring. Complete deprotection was assessed by RP-HPLC–MS analysis (Figure. S2 panel B). A shift of the retention time (RT) from 26.74 to 26.32 min was observed upon sugar deprotection, due to the higher hydrophilic character of the fully deprotected product with respect to its precursor.

### Peptide purification

Peptide purification was accomplished using a Shimadzu LC-8A preparative HPLC system (Shimadzu, Kyoto, Japan), equipped with an SPD-M10AV UV–Vis detector. A Reverse Phase (RP) Vydac C18 column (250 cm × 22 mm; 10 μm) was eluted with a linear gradient of H_2_O 0.1% TFA (eluent A) and acetonitrile 0.1% TFA (eluent B), at 23 mL/min. A gradient from 10 to 60% B over 30 min was used for LL-III, while a slower gradient (from 30 to 50% B over 15 min) was used to achieve a good separation from undesired byproducts in the case of g-LL-III. Peptide purity and identity were assessed by RP-HPLC–MS analyses (Figure. S3, S4, S5 and S6), using a Shimadzu LC-10ADvp equipped with an SPDM10Avp diode-array detector. Mass spectra were recorded on a Shimadzu LC–MS-2010EV system with ESI interface and a quadrupole mass analyzer. A Vydac C18 column (150 mm × 4.6 mm, 5 μm) was eluted with the same gradients used for purification, over 30 min at a flowrate of 1 mL/min in the LC analyses and over 60 min at a flowrate of 0.5 mL/min in the LC–MS analyses. ESI–MS analyses were performed in the positive ion scanning mode, in the 400–1800 Th range of m/z. The trifluoroacetate counterion was removed from the synthesized peptides by anionic exchange with chloride. The pure peptides (2 mg/mL) were dissolved in 50 mM HCl, then frozen and lyophilized after 5 min incubation. The procedure was repeated twice. Complete removal of TFA was assessed by ^19^F NMR (data not shown).

### Vesicles preparation

Vesicles were prepared by the thin-film hydration method as described before^34^ to obtain a suspension of multilamellar vesicles (MLVs). Large unilamellar vesicles (LUVs) of 100 nm size were obtained by extruding MLVs at least 21 times through two stacked polycarbonate filters and a permeable membrane with 100 nm pores (Nuclepore, Pleasanton, CA, USA). The whole procedure was carried out using an extruder (Avanti Polar Lipids Inc. Alabaster, AL, USA) filled with two 1.0 mL Hamilton syringes (Hamilton, Reno, NV, USA). Vesicles size was confirmed by means of dynamic light scattering (DLS) measurements, which showed a mean hydrodynamic radius consistent with the formation of LUVs. Liposomes with different lipid composition were prepared: POPC/POPG (8/2 mol/mol) and DPPC/POPG (8/2 mol/mol). Liposome samples in the presence of peptides were prepared by mixing appropriate amounts of peptide solution and liposomes suspensions to obtain the required lipid-to-peptide (L/P) ratio. The vesicles containing the fluorescent probe Laurdan were obtained by adding a solution of Laurdan in DMF to the lipid organic mixture at a lipid/Laurdan mole ratio of 30, while vesicles containing the fluorescent probe DPH were obtained by adding a solution of DPH in chloroform to the lipid organic mixture at a lipid/DPH mole ratio of 150.

### Circular dichroism spectroscopy (CD)

CD measurements were performed on a Jasco J-1500 Spectropolarimeter (Jasco Analytical Instruments, Tokyo, Japan). The spectra were recorded at 25 °C in a 0.1 cm quartz cuvette and 190–260 nm interval range with 0.5 nm resolution, 50 nm/min scanning speed, 4 nm bandwidth and 2 s integration time. The spectra were averaged over five accumulations and normalized per mole of residue. CD spectra were recorded in the absence and presence of POPC/POPG LUVs at a total lipid concentration of 750 µM (L/P ratio of 50). For each sample, a background blank was subtracted.

### Differential scanning calorimetry (DSC)

The heat capacity curve of DPPC/POPG MLVs was recorded in the absence and presence of both peptides, using a high sensitivity Nano DSC (TA Instruments, New Castle, DE, USA) equipped with 300 µL twin gold capillary cells, pressurized to 3 atm. Each sample was scanned from 20 to 55 °C at least four times, using a scanning rate of 1 °C/min. For each thermogram, a background blank thermogram was subtracted. Data were analyzed using the Nano Analyze software, provided by the manufacturer. Enthalpy values were obtained by direct integration of the normalized baseline subtracted peaks.

### Fluorescence spectroscopy

Fluorescence experiments were performed on a Fluoromax-4 (Horiba, Edison, NJ, USA) operating in the steady state mode at the temperature of 25 °C.

#### Binding experiments

The interaction of peptide with liposomes was studied by monitoring the Trp fluorescence emission spectra at increasing POPC/POPG lipid concentration. The peptide concentration was fixed at 7 µM, while the total lipid concentration ranged between 0 and 1 mM. Emission spectra were recorded between 300 and 450 nm after excitation at 280 nm. The binding isotherm was obtained by plotting the relative fluorescence intensities (*F*/*F*_0_) at 329 nm as a function of lipid concentration, where F_0_ and F is the fluorescence intensity of the peptide in the absence and presence of vesicles, respectively. The reported data were analyzed to obtain the mole fraction partition constant (*K*_x_)^35^.

#### Fluorescence anisotropy

Fluorescence anisotropy of the Laurdan and DPH probes embedded in POPC/POPG LUVs were monitored at 476 nm and 425 nm, respectively. The excitation wavelengths were set to 340 nm and 355 nm for Laurdan and DPH, respectively. All the measurements were performed in a 1 cm path length quartz cuvette. The total lipid concentration was fixed at 50 µM, while the peptide concentration ranged between 0 and 50 µM. The fluorescence anisotropy (< *r* >) was determined according to the relation < *r* >  = (*I*_VV_* − GI*_VH_)/(*I*_VV_ + 2*GI*_VH_), where *I*_VV_ is the fluorescence intensity obtained by setting both the excitation and emission polarizers vertically, *I*_VH_ is the fluorescence intensity obtained by setting the excitation polarizer vertically and the emission polarizer horizontally and *G* is the instrument-specific correction factor^36^.

#### Laurdan fluorescence emission spectra and GP calculation

Laurdan fluorescence emission spectra were recorded in the 360–650 nm range, using an excitation wavelength of 340 nm. Both slits for the excitation and emission monochromators were set to 7 nm. Measurements were performed in a 1 cm path length cuvette. The total lipid concentration was fixed at 50 µM, while the peptide concentration was varied between 0 and 50 µM. The Laurdan concentration was 1.5 µM. Laurdan generalized polarization (*GP*) was calculated according to the relation *GP* = (*I*_440_* − I*_490_)/(*I*_440_ + *I*_490_), where *I*_440_ and *I*_490_ are the fluorescence intensity at 440 and 490 nm, respectively^37^.

#### Fluorescence kinetics

Fluorescence kinetics experiments were performed to evaluate the resistance of both g-LL-III and LL-III against enzymatic cleavage, using the substrate AAF-AMC and α-chymotrypsin and pepsin as enzymes. The concentrations of peptides were fixed at 50 μM, of the substrate AAF-AMC at 80 μM, and of α-chymotrypsin and pepsin at 10 nM and 5 μM, respectively. The substrate AAF-AMC was dissolved in DMSO. The stock solution of α-chymotrypsin was prepared in phosphate buffer at pH 7.4 and their concentrations were evaluated as previously described^38^. The pepsin stock solution was prepared in acetate buffer at pH 4.0 and its concentration was evaluated spectrophotometrically using a molar extinction coefficient of 49.650 cm^−1^ M^−1^^39^. Experiments were performed in a 0.3 cm pathlength cuvette, monitoring the fluorescence intensity at 460 nm with slits for the excitation and emission monochromators set to 7 nm.

#### Time-resolved fluorescence spectroscopy of DPH

Time-resolved fluorescence experiments of DPH embedded in POPC/POPG LUVs were performed by means of a K2 multi-frequency phase fluorimeter from ISS (Champaign, IL, USA) using the frequency domain method (frequency range 2–100 MHz), where the intensity of the excitation light was sinusoidally modulated and the phase shift of the fluorescence, relative to the excitation, is determined. The wavelength of excitation was set to 370 nm using a diode light source. The fluorescence emission was collected through a 395-nm long pass filter. As standard for lifetime determination, dimethyl-POPOP was used, which is characterized by a lifetime of 1.45 ns in ethanol. The experiments were performed at the temperature of 25 °C. The obtained frequency responses plots were analyzed by means of Vinci software provided by the manufacturer.

### Biological assays

#### Antimicrobial activity and analysis of peptide stability

The antimicrobial activity of g-LL-III and LL-III was tested against a panel of both *Gram-negative* and *Gram-positive* bacteria (*Escherichia coli* ATCC 25,922, *Pseudomonas aeruginosa* 01, *Acinetobacter baumannii* ATCC 17,978, *Klebsiella pneumoniae* ATCC 700,603 *Staphylococcus aureus* ATCC 29,213, *Bacillus subtilis* 6633, MRSA WKZ-2 and *Bacillus globigii* TNO BMO13 as previously described^40^. Briefly, bacteria were grown to midlogarithmic phase in Luria Bertani broth (LB) at 37 °C. Cells were then diluted to 1 × 10^6^ CFU/mL in Difco 0.5X Nutrient Broth (Becton-Dickenson, Franklin Lakes, NJ) containing increasing amounts of peptide. Starting from a peptide stock solution, two-fold serial dilutions were sequentially carried out, accordingly to the broth microdilution method^41^. Following over-night incubation, MIC_100_ values were determined as the lowest peptide concentration needed for no visible bacterial growth. As control, the antimicrobial activity of ciprofloxacin has been evaluated on all tested strains.

To analyze the stability of the peptides to the proteases, 100 μM of LL-III and glycosylated LL-III were incubated with 100 nM of α-chymotrypsin or pepsin at 37 °C for 15 min and then antimicrobial activity were assayed as reported above. The peptide solution without proteases was used as the control group.

Finally, to determine peptides’ serum stability, a 200 μM solution of g-LL-III or LL-III were incubated with fetal bovine serum (FBS) in a 1:1 ratio (v/v) for 24 h at 37 °C, and the antimicrobial activity was determined as described above.

#### Cell viability

The cytotoxicity of g-LL-III and LL-III was measured on Simian-virus-40-transformed mouse fibroblasts (SVT2 cells) and the parental non- transformed BALB/c 3T3 cell lines. In brief, 5·10^3^ cells were seeded on a 96-well plate and after 16 h treated with increasing concentrations of peptides for 24 h at 37 °C and 5% CO_2_. Cell viability was analyzed by performing the (3-(4,5-dimethylthiazol-2-yl)-2,5 diphenyltetrazolium bromide) reduction inhibition assay (MTT assay)^42^.

## Results and discussion

### Chemically modified LL-III

The antimicrobial peptide LL-III was chemically modified by replacing the Asn residue, with the N-acetylglucosamine (NAG) substituted amino acid, obtaining the N-glycosylated form of LL-III, denoted g-LL-III. The sugar moiety and its position in the sequence were chosen considering that: (i) the NAG is a carbohydrate naturally occurring in many proteins and peptides undergoing post-translational modifications, (ii) in several AMPs subjected to glycosylation, the Asn residue is a site of modification: in the LL-III sequence, it is the only residue that can be glycosylated^30^, (iii) the Asn is the second residue starting from the N-terminus, thus offering a natural capping position of that region of the peptide (instead, the C-terminus is amidated). A complete characterization of the interaction of g-LL-III with liposomes, composed of POPC/POPG (8/2 mol/mol) mimicking the basic bacterial membrane, is reported in the following section. The results were compared with data previously obtained for the parent peptide LL-III, using the same model of negatively charged membrane. The stabilities of the peptides to the proteolytic enzymes α-chymotrypsin and pepsin were then determined by both a substrate competition and an antimicrobial assay. Further, bioactivities of LL-III and g-LL-III have been analyzed against several bacterial strains and two eukaryotic cells lines. Finally, the stability of g-LL-III and LL-III has been also tested *in serum*.

### Probing the interaction of g-LL-III with bacterial model membranes

**Figure 1:**
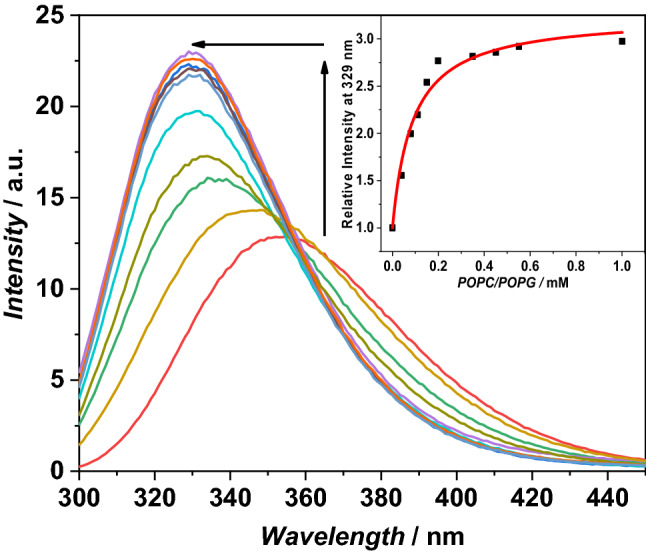
Fluorescence emission spectra of g-LL-III at increasing lipid concentration. The arrows indicate the increase of fluorescence and the blue shift of the position of the maximum. Inset: binding isotherm obtained by plotting the relative fluorescence intensity at 329 nm *vs. *lipid concentration. The red line is the best fit of experimental data (please, refer to the Materials and Methods section for details). The experiments were performed using LUVs, 10 mM sodium phosphate buffer, pH 7.4, 25 °C.

The first step of antimicrobial peptides mechanism of action is the interaction with the lipid bilayer of pathogens^14^. We evaluated the interaction of g-LL-III with model membranes by determining the mole fraction partition constant, *K*_x_. The *K*_x_ value was obtained by titrating a solution of g-LL-III, at fixed concentration of 7 µM, with a suspension of POPC/POPG vesicles, and following changes in the Trp3 fluorescence. Figure 1 shows the collected fluorescence emission spectra of g-LL-III in the absence and presence of increasing lipid concentration (in the range 0–1 mM).

The emission spectrum of the g-LL-III peptide in neat buffer is centered at about 353 nm, according to a well exposed Trp residue to the aqueous solvent, as observed for various linear cationic (and unstructured) peptides^43–45^. Upon addition of POPC/POPG vesicles, a marked blue shift (approximately 23 nm at 1 mM POPC/POPG) coupled with an increase in emission intensity was observed. These characteristics indicate that the peptide interacts with the membrane and experiences a more hydrophobic and constrained environment. By fitting the relative fluorescence intensity at 329 nm *vs.* the total lipid concentration curve (see inset of Fig. 1) the partition constant *K*_x_ = (7.0 ± 1.0)∙10^5^ was calculated. A similar behavior was previously observed for the parent peptide, LL-III, which showed a slightly lower a partition constant of (4.5 ± 1.0)∙10^5^ in the same experimental conditions^21^. All these data clearly indicate that the glycosylation has no significant effect on the interaction of the peptide with the bacterial model membrane.

### Conformational changes of g-LL-III

**Figure 2:**
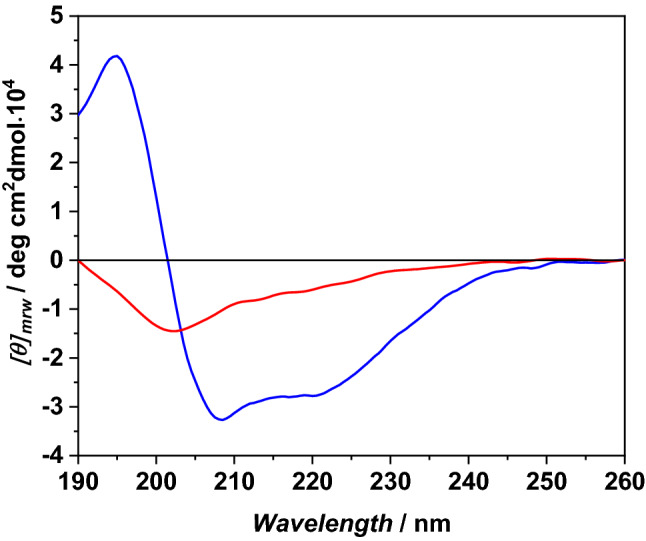
CD spectra of g-LL-III in the absence (red line) and presence of POPC/POPG LUVs at L/P 50 ratio (blue line). The spectra were acquired using a 0.1 cm path length quartz cuvette, in 10 mM phosphate buffer at pH 7.4 and temperature 25 °C.

It is well known that most linear, unstructured, AMPs adopt a helical conformation upon binding to membranes^14,46^.This folded structure seems fundamental to AMPs to exert their biological function ^9,47^. The conformational behavior of g-LL-III in the absence and presence of POPC/POPG vesicles was investigated by circular dichroism (CD) spectroscopy. Figure 2 reports the far-UV CD spectra of g-LL-III in neat buffer and in the presence of POPC/POPG vesicles at the lipid-to-peptide ratio, L/P = 50.

The CD spectra clearly show that the conformation of g-LL-III adopted in solution strongly depends on the presence of lipid vesicles. In neat buffer solution, the peptide adopts an unordered structure as indicated by the presence of a negative band around 200 nm. In the presence of vesicles, the CD spectrum is consistent with an α-helical conformation with its characteristic minima at 208 nm and 222 nm as well as by the maximum at 195 nm. Similar results have been reported for LL-III with the same model membranes^21^. Collectively, these results show that the presence of the sugar moiety attached to the Asn residue of LL-III does not hamper the conformational change imposed by the peptide-vesicle interaction.

### Membrane perturbation induced by the g-LL-III

#### Perturbations of the bilayer hydrophobic core

**Figure 3:**
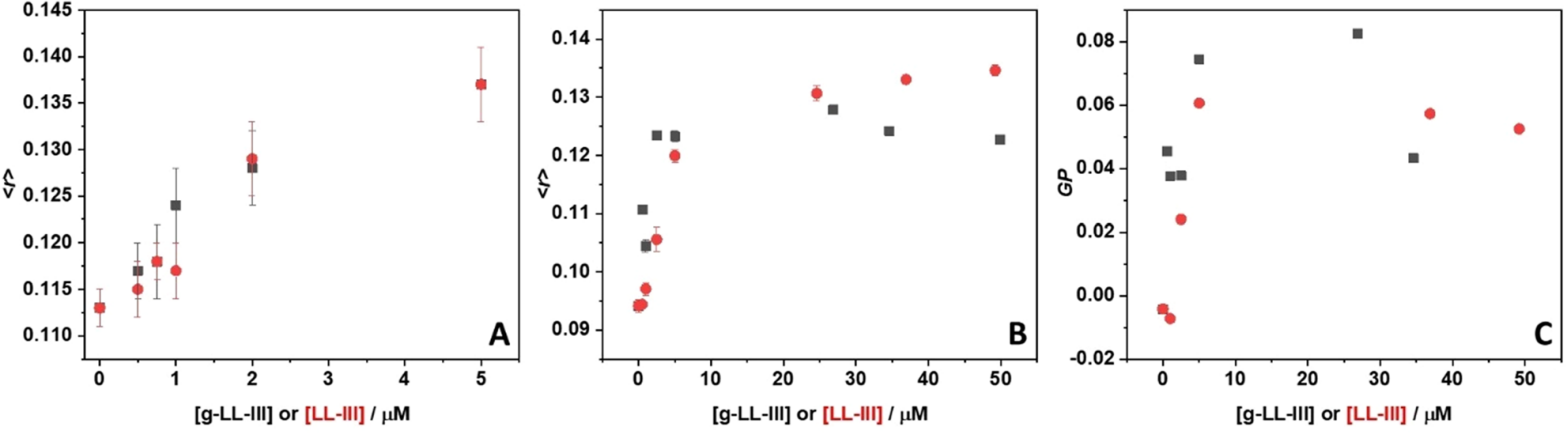
Fluorescence anisotropy (< *r* >) of the probes (**A**) DPH, (**B**) Laurdan and (**C**) generalized polarization (*GP*) of Laurdan embedded in POPC/POPG vesicles in the presence of g-LL-III (black squares) and LL-III (red circles). All the experiments were performed at 25 °C in 10 mM phosphate buffer, pH 7.4.

Steady-state fluorescence anisotropy experiments of the probe DPH in POPC/POPG vesicles were performed to yield information on the peptide abilities to perturb the bilayer hydrophobic core. DPH is a probe that partitions inside the hydrophobic core of the membrane serving as reporter group of that region of the bilayer^48^. Figure 3A shows the static fluorescence anisotropy data of DPH as a function of g-LL-III and, for comparison, LL-III concentrations. The anisotropy, < *r* > , of DPH embedded in POPC/POPG vesicles in the absence of peptides is around 0.11, a value compatible with a bilayer in the fluid-like (liquid-disordered) lipid phase^21^. Upon addition of g-LL-III peptide, a concentration dependent increase of < *r* > was observed, reaching a value round 0.137 at 5 µM of peptide (L/P = 10). Even though increase of < *r* > is quite small, this result indicates that the peptide hampers the rotational motion of DPH, *i.e.*, addition of g-LL-III leads to an increase of the order parameter of the lipid chains, indicating interaction of the peptide essentially with the surface of the lipid bilayer. A similar result was obtained for the non-glycosylated LL-III, thereby indicating the same kind of interaction for both peptides.

#### Perturbation of the head group region of the lipid bilayer

Using Laurdan as fluorescent probe, it is possible to study the effect of g-LL-III and LL-III at the water/membrane interface, *i.e.,* the head group region of the lipid bilayers. Laurdan positions itself at the water–lipid headgroup interface, so that fluorescence anisotropy measurements elucidate how the lipid packing is affected by the addition of the peptides in this region of the membrane. Figure 3B shows the steady-state anisotropy of Laurdan embedded in POPC/POPG unilamellar vesicles in the absence and in the presence of increasing concentrations of g-LL-III. The value of ⁓0.095 for the anisotropy of Laurdan embedded in POPC/POPG LUVs indicates that the bilayer is in the liquid-disordered phase, as suggested by the DPH experiments. Upon addition of g-LL-III, a concentration dependent increase of < *r* > was observed. In the range 0–5 µM, an almost linear increase of < *r* > was found, and at concentrations beyond 5 µM, the anisotropy did not change further, indicating that saturation was reached. The increase of < *r* > is a clear indication that upon binding to the lipid bilayer, g-LL-III is inducing a higher degree in the head groups regions, leading to a more compact lipid packing of the membrane. This effect may propagate inside the membrane, leading to an overall rigidification of the bilayer. Remarkably, the same effect was observed (qualitatively and quantitatively) for the parent unglycosilated peptide. Additional information about the hydration state at the membrane/water interface can be obtained from steady state fluorescence emission spectra of Laurdan^37^. The Laurdan emission spectrum is composed of two well resolved bands, which arise from two different emitting states: a non-solvent and a solvent relaxed state. The emission band from the non-solvent relaxed state (around 440 nm) is due to the Laurdan molecules embedded in a less hydrated gel-phase. Conversely, the emission band from the solvent relaxed state (at around 490 nm) is due to Laurdan localized in a more hydrated, and less packed liquid-disordered phase^37,49^. To quantitatively describe the spectroscopic signatures of Laurdan, the general polarization (*GP*) can be used^50^, which is defined as:

$$GP = \frac{{I_{440} - I_{490} }}{{I_{440} + I_{490} }}$$

where *I*_440_ and *I*_490_ are the fluorescence intensities of Laurdan at 440 nm (non-solvent relaxed) and at 490 nm (solvent relaxed), respectively. The *GP* value can adopt any value between + 1 and − 1. According to the equation reported above, when the membrane is in the more packed, less hydrated state, the emission from the non-solvent relaxed state dominates (*I*_440_ > *I*_490_) and the *GP* assumes more positive values respect to a membrane in the liquid disordered phase (where *I*_440_ < *I*_490_). Figure 3C depicts the *GP* values of Laurdan embedded in POPC/POPG vesicles at increasing concentrations of g-LL-III or LL-III. Similar to the anisotropy values, for both peptides, an almost linear increase was observed in the range 0–5 µM. At concentrations higher than 5 µM, *GP* does not change anymore. The increase of *GP* evidences that both peptides similarly induce dehydration of the membrane surface. Thus, we can conclude that the presence of the covalently attached sugar moiety does not alter the ability of the peptide to bind to the lipid bilayer interface.

#### The LL-III peptides induce lipid domains formation

**Figure 4:**
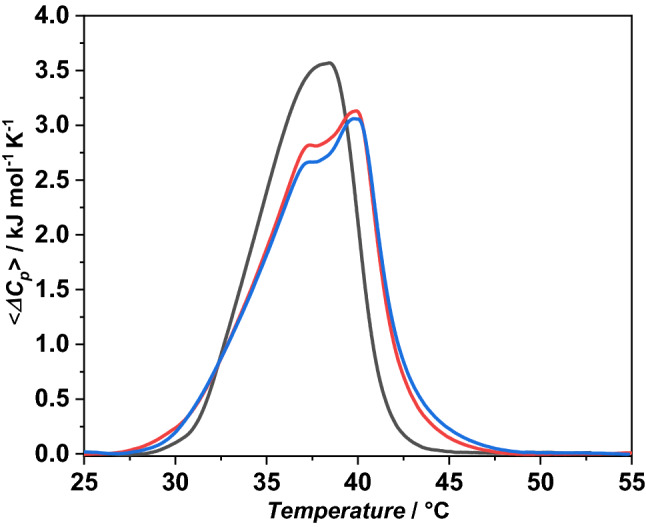
(**A**) DSC traces of DPPC/POPG multilamellar vesicles in the absence (black line), and in the presence of g-LL-III (red line) and LL-III (blue line) at the L/P 10. The experiments were performed in 10 mM phosphate buffer, pH 7.4.

Differential scanning calorimetry (DSC) is a powerful tool to study thermotropic structural phase transitions of lipid bilayers and how they are affected by the interaction with exogenous molecules, such as peptides and proteins^43,51–53^. DSC measurements were performed to investigate the changes of thermotropic structural changes of a suitable model membrane induced by the interaction of g-LL-III. To this end, the POPC/POPG system, which exhibits a gel-to-liquid phase transition temperature around –5 °C, was replaced by a DPPC/POPG mixture to achieve vesicles with a more suitable chain-melting transition temperature. Figure 4 shows the DSC profiles of DPPC/POPG vesicles in the absence and presence of both g-LL-III and LL-III at the lipid-to-peptide ratio of 10. Table 1 summarizes the thermodynamic parameters obtained from the DSC analysis.

**Table 1:** Thermodynamic parameters for the gel-to-liquid phase transition of DPPC/POPG (8/2 mol/mol) MLVs, in the absence and presence of g-LL-III and LL-III at L/P = 10.

System	*T*_*m*_/°C	^a^Δ*H*_*m*_/kJ mol^−1^
DPPC/POPG	38.1 ± 0.1	23.4 ± 1.1
+ g-LL-III	37.5 ± 0.1–39.9 ± 0.1^b^	24.0 ± 1.2
+ LL-III	37.5 ± 0.1–39.9 ± 0.1^b^	24.2 ± 1.2

^a^Normalization against total lipid moles. ^b^The two indicated *T*_*m*_ values refer to the first and second maxima in the thermogram.

The thermogram of DPPC/POPG vesicles (Fig. 4, black line) is characterized by a transition temperature at ⁓38 °C, with an enthalpy change of 23.4 kJ mol^−1^. It is important to note that DPPC and POPG lipids are fully miscible^54^, thus the thermogram represents the gel-to-liquid phase transition of the DPPC bilayer containing a uniform distribution of POPG. Upon addition of the g-LL-III, a dramatic change in the DSC peak was observed. The thermogram is now composed by two well-defined and separated transitions, one centered at about 37.5 °C and a second one at ~ 40 °C. This result strongly evidences microheterogeneities within the bilayer, due to the formation of lipid domains induced by the peptide binding. Most likely, a preferential interaction of the cationic peptide with the negatively charged POPG occurred, leading to the formation of domains that melt at different temperature. The DSC peak at higher temperature is expected to represent melting of a DPPC-enriched domain with a chain-melting temperature close to that of pure DPPC bilayers, while the peak at the lower temperature likely represents the PG-enriched domain, still containing some percentage of DPPC molecules. Inspection of Table 1 shows that the enthalpy change is only slightly affected by the peptide, thus indicating that the overall packing among lipid chains is largely preserved and, thus, that the peptide is unable to penetrate the interior of the hydrophobic core of the membrane^45^. Upon addition the parent peptide LL-III, similar results were obtained, in excellent agreement with previously reported data, where the domain formation was also observed through DSC experiments on DPPC/DPPG vesicles^21^. In conclusion, glycosylation of the peptide has no significant effect on the formation of lipid domains induced by addition of the peptide.

**Figure 5:**
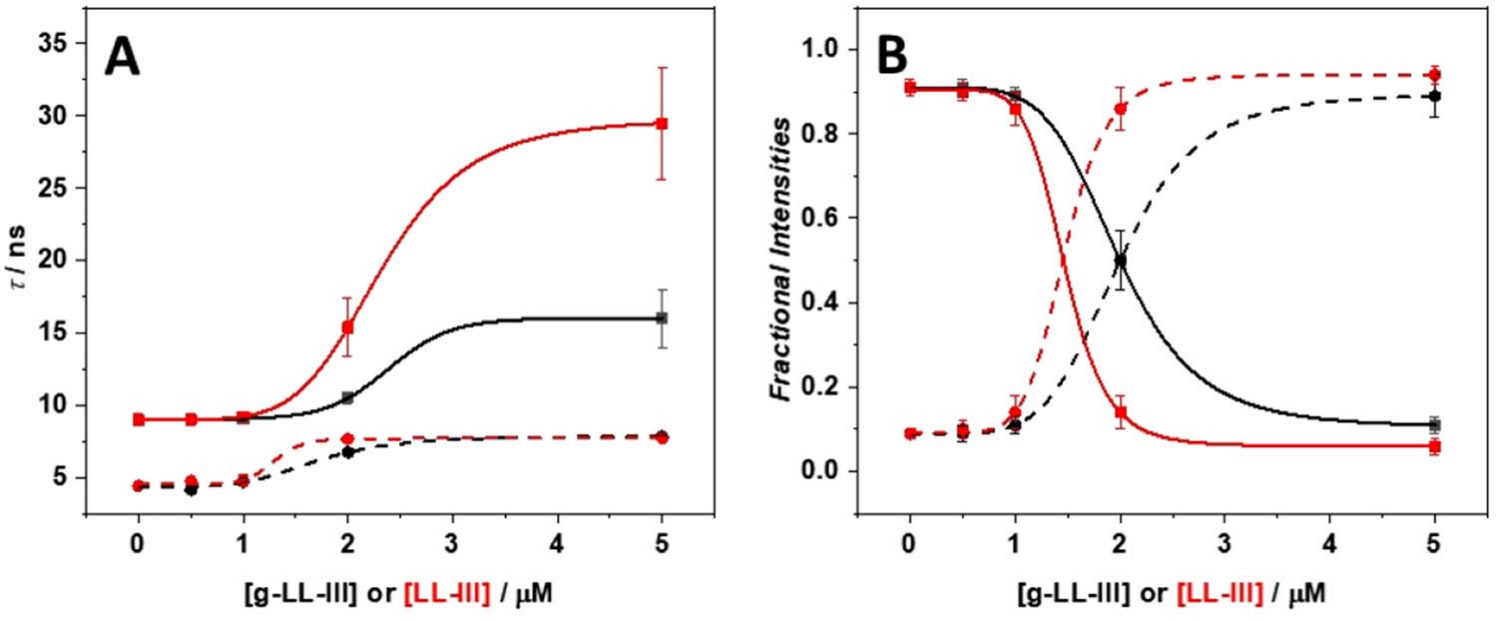
Fluorescence lifetimes (**A**) and fractional intensities (**B**) of DPH embedded in POPC/POPG LUVs in the absence and presence of g-LL-III and LL-III peptides, at the indicated concentrations. In panel A, squares and solid lines refer to τ_1_, while circles and dashed lines refer to τ_2_. In panel B, squares and solid lines refer to *f*_1_, while circles and dashed lines refer to *f*_2_. Experimental data referring to g-LL-III and LL-III are reported in black and red, respectively. Lines are a guide for the eyes, only.

To gain further insights into the ability of the g-LL-III peptide to induce lipid domains formation within the bilayer, time-resolved fluorescence experiments were performed using the DPH probe embedded in POPC/POPG bilayer. Figure S7 shows the measured phase delay and modulation ratio of DPH in POPC/POPG vesicles in the absence and presence of g-LL-III and LL-III at L/P = 10 as a function of the frequency of the excitation intensity modulation. Figure 5 depicts the fluorescence lifetimes of DPH in the absence and presence of peptides at different L/P ratios at 25 °C.

Two different values of decay times were observed for the DPH fluorescence in POPC/POPG at 25 °C, where the bilayer is in the liquid crystalline phase: a higher one (τ_1_ = 9.01 ns) with a fractional intensity (*f*_1_) around 0.91, and a lower one (τ_2_ = 4.42 ns) with an associated fractional intensity (*f*_2_) of 0.09. These values are in agreement with previously reported values for a bilayer in the liquid phase, such as POPC vesicles^55^. These two decay times have been attributed to environmental heterogeneities within the bilayer^56^. Indeed, the decay time of DPH is sensitive to the dielectric constant, which changes along the normal of the bilayer surface, and is largely controlled by the level of hydration. With increasing concentrations of g-LL-III, an increase of both τ_1_ and τ_2_ were observed with a concomitant decrease of *f*_1_ and increase of *f*_2_. Indeed, at the highest peptide concentration (L/P = 10), τ_1_ is ⁓16 ns, τ_2_ is ⁓7.9 ns with the associated fractional intensities of 0.11 and 0.89 respectively. This behavior could be justified as follows. At high peptide concentration (L/P = 10), as inferred by DSC experiments, the domains formation process takes place. The highest decay time, with a *f*_1_ = 0.11 could be associated to the PG enriched domains where the peptide is bound. The bound peptide can strongly rigidify these domains leading to a marked increase of τ_1_ (⁓16 ns). This increase is due to the strong reduction of all the excited-state deactivation process upon domains formation (*e.g.*, those induced by quenching through water molecules). In addition, since the amount of POPG is much lower compared to POPC, the PG domains represent a small fraction of the environment where the DPH is embedded (hence, low value of *f*_1_ = 0.11). The other PCs enriched domain represents instead the larger fraction of the environment where DPH is localized (*f*_2_ = 0.89). Some peptide molecules are, obviously, bound to these domains too, leading to the increase of the decay time (τ_2_ = around 7.9 ns). Thus, combining DSC data with fluorescence lifetime measurements, it is possible to demonstrate that g-LL-III peptide is able to induce the formation of small microdomains in the bilayer through a preferential (surface) interaction with PG lipids. Similar results were obtained for the parent peptide LL-III. However, at L/P = 10 the increase in τ_1_ and the decrease in *f*_1_ are more prominent. This could be ascribed to a higher propensity of LL-III to prompt formation of densely packed domains, suggesting that the presence of sugar moiety has some effect on the interaction process, although this effect is likely to be small.

Collectively the reported results indicate that the g-LL-III peptide, interacting preferentially with the negatively charged PGs, is able to prompt the formation of lipid domains within the bilayer without penetrating in the hydrophobic core of the membrane. The comparison with the parent peptide LL-III reveals that the presence of the sugar moiety does not affect the capability of the peptide to perturb the membrane integrity markedly. The presence of lipid domains highlights that both peptides are able to cause redistribution of lipid molecules within the bilayer plane, which is expected to impact the cell’s viability. The domains formed can be characterized by a different fluidity and packing density, which in turn leads to malfunction of fundamental membrane-associated processes and reactions. Moreover, the interface between domains can be considered as “hot spot” through which the peptide can gain access to the intracellular space targeting proteins and nucleic acids, which affects the viability of the cell. Indeed, translocation across the membrane and localization near mitochondria and nucleoli in tumor cells was demonstrated for LL-III^18^.

### Effect of glycosylation on peptides hydrolysis

**Figure 6:**
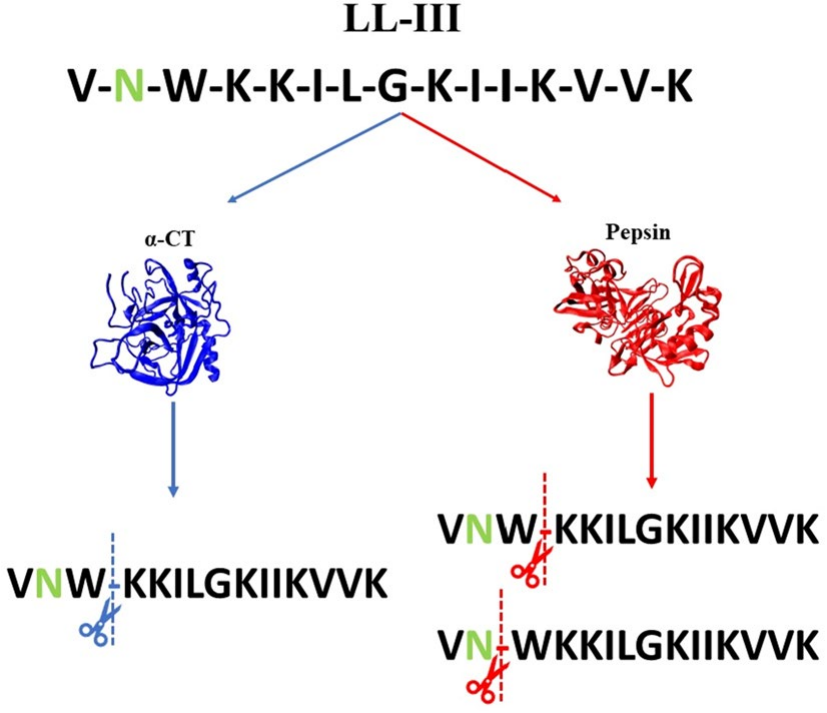
Hydrolysis sites of peptide bonds in LL-III peptide catalyzed by α-CT (right side) and pepsin (left side). The enzyme α-CT hydrolyzes the peptide bond at the C-terminus of the Trp residue (position 3). Instead, pepsin acts at the level of positions 2 and 3. The Asn residue is highlighted in green, to which NAG is covalently linked. The structures of α-CT (pdb code: 1ACB) and pepsin (pdb code: 4PEP) were drawn using VMD software^62,63^. The prediction of hydrolysis sites on the peptides was performed with PeptideCutter software^58^.

As stated in the Introduction section, the final goal of the glycosylation is to render the LL-III more resistant to the enzymatic hydrolysis. To this end, a sugar (NAG) moiety was covalently bound to the Asn residue of the peptide. To test the resistance of the peptides against enzymatic hydrolysis, in vitro assays with two enzymes, α-chymotrypsin (α-CT) and pepsin, were performed. The enzyme α-CT is a proteolytic enzyme of the mammalian digestive system, active at neutral pH, that selectively hydrolyze the peptide bond at the C-terminus of aromatic residues^38,57,58^. In the present case, α-CT hydrolyzes the peptide bond at position 3, at the C-terminus of the Trp residue (see Fig. 6). Pepsin is an endopeptidase, active at acidic pHs, also present in the digestive system of humans and other animals^59^. The hydrolysis of peptide bond by pepsin depends on the residues close to the cutting site^58,60,61^. In the present case, pepsin hydrolyzes LL-III at position 2 and 3 (Fig. 6), in proximity of the Trp residue.

**Figure 7:**
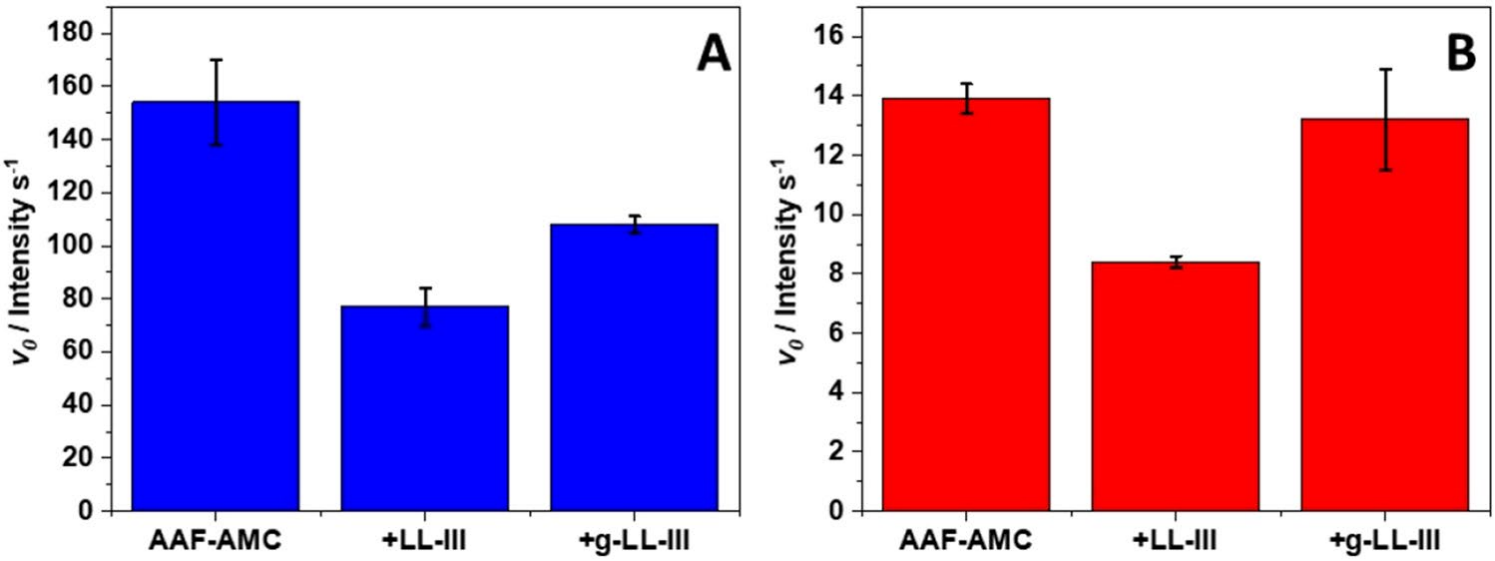
Initial rates (*v*_0 _/ *Intensity s*^*−1*^) for the hydrolysis of the substrate AAF-AMC catalyzed by (**A**) α-chymotrypsin at pH 7.4 and (**B**) pepsin at pH 4.0, in the absence and in the presence of LL-III and g-LL-III peptides. The concentration of α-chymotrypsin and pepsin were 10 nM and 5 µM, respectively. The concentration of the fluorescent substrate AAF-AMC was 80 µM, and the concentration of both peptides were fixed at 50 µM. All the experiments were performed at 25 °C.

The experiments were performed at pH 7.4 and 4.0, for α-CT and pepsin, respectively. The substrate used to verify the effect of glycosylation was the tripeptide Ala-Ala-Phe-7-amido-4-methylcoumarin (AAF-AMC). The hydrolysis of the bond between Phe and AMC can be followed by exciting it at 370 nm and recording the fluorescence emission at 460 nm as function of time, without interferences from the substrate. To quantitatively evaluate the kinetics of release, the slope of the linear portion of the fluorescence *Intensity vs. time* plot was determined (Figure S8 shows the kinetic curves for α-CT). The peptides g-LL-III and LL-III act as competitive substrates for AAF-AMC. Consequentially, if a peptide is hydrolyzed by the enzymes, the kinetics of release of AMC from AAF-AMC should be retarded. Conversely, if a peptide is not hydrolyzed at all, the kinetics of release of AMC should be similar to that of the control (*i.e.,* in the absence of g-LL-III or LL-III). Considering different affinities of the two enzymes for AAF-AMC and the peptides, different responses may be expected. Figure 7 reports the initial rates, *v*_0_, in the absence and presence of g-LL-III and LL-III for the hydrolysis of AAF-AMC catalyzed by α-CT (panel A) and pepsin (panel B).

For the hydrolysis of AAF-AMC catalyzed by α-CT at pH 7.4, a slope of (154 ± 16 *Intensity s*^*−1*^) was obtained. Upon addition of 50 µM of LL-III, a marked decrease of *v*_0_ was observed (now 77 ± 7 *Intensity s*^*−1*^), indicating that LL-III is bound and its peptide bond neighboring the Trp residue is hydrolyzed. In the presence of g-LL-III at the same concentration, the slope is significantly higher (108 ± 3 *Intensity s*^*−1*^), signifying that the presence of NAG partially hampers the complex formation between α-CT and the peptide and, thus, is less prone to the hydrolysis respect to the parent peptide. For pepsin, the hydrolysis of AAF-AMC shows a slope of (13.9 ± 0.5 *Intensity s*^*−1*^). In the presence of LL-III, the slope drops to (8.4 ± 0.2 *Intensity s*^*−1*^), indicating again that LL-III is a competitive substrate for AAF-AMC and glycosylation of LL-III leads to similar values compared to the control (13.2 ± 1.7 *Intensity s*^*−1*^). These results demonstrate that the NAG moiety is able to strongly hamper the binding of the peptide to the hydrolyzing enzyme. In other words, the presence of the NAG group effectively increases peptide’s stability against protease degradation.

### Peptides antimicrobial activity and stability

The antimicrobial activity of LL-III, g-LL-III and ciprofloxacin (control) was evaluated on a panel of both *Gram-negative* (*Escherichia coli* ATCC 25,922, *Pseudomonas aeruginosa* 01, *Acinetobacter baumannii* ATCC 17,978 and *Klebsiella pneumoniae* ATCC 700,603) and *Gram-positive* (*Staphylococcus aureus* ATCC 29,213, *Bacillus subtilis* 6633, MRSA WKZ-2 and *Bacillus globigii* TNO BMO13) bacterial strains. Increasing doses of g-LL-III and LL-III (50–0.78 μM) were administrated to 1 × 10^6^ CFU/mL of each strain and their antimicrobial properties were measured determining their minimal inhibitory concentrations (MICs). The MIC values are collected in Table 2. The data highlight that LL-III is a peptide characterized by low MIC values indicating that this peptide is particularly active, in agreement with previously reported data^16^. Overall, the comparison between the g-LL-III and LL-III clearly indicates that the LL-III glycosylation procedure does not significantly affect the peptide’s powerful antimicrobial activity. However, the standard antibiotic ciprofloxacin is more active respect to the tested peptides, as it shows MIC values that are between twenty- and two-times lower respect to that obtained for the peptides (see Table 2). A slight difference between the two peptides was observed for *P. aeruginosa* 01. Indeed, the MIC value of g-LL-III is two times higher respect to the value obtained for LL-III, indicating a reduced activity of the glycosylated form. This behavior could be explained by the higher hydrophobicity of LL-III: the introduction of the sugar moiety led to the more hydrophilic g-LL-III peptide. Indeed, a correlation between hydrophobicity and antimicrobial activity against several *P. aeruginosa* strains was previously observed for V13K_L_ peptide^64^. In addition, the presence of NAG could induce small conformational changes of the peptide and, thus, influence its ability to interact with the bacterial membranes, which, it should be noted, may strongly differ in composition from strain to strain^26,65^.

**Table 2:** Minimal inhibitory concentrations (MIC, in µM) of g-LL-III and LL-III against the reported bacterial strains.

MIC/µM			
Bacterial strains	Peptides		Standard antibiotic
	LL-III	g-LL-III	Ciprofloxacin
*Escherichia coli* ATCC 25,922	3.12	3.12	0.15
*Pseudomonas aeruginosa* 01	1.56	3.12	0.3
*Acinetobacter baumannii* ATCC 17,978	3.12	3.12	0.15
*Klebsiella pneumoniae* ATCC 700,603	3.12	3.12	1.2
*Staphylococcus aureus* ATCC 29,213	1.56	1.56	0.6
*Bacillus subtilis* 6633	1.56	1.56	0.15
MRSA WKZ-2	3.12	3.12	0.3
*Bacillus globigii* TNO BMO13	1.56	1.56	0.3

To better support these previous findings on the increased resistance of g-LL-III toward proteases, we analyzed the consequences of the proteolytic activity of α-chymotrypsin and pepsin also on the antimicrobial activity of both LL-III and g-LL-III against *Escherichia coli*. Both peptides were pretreated with the proteases for 15 min at 37 °C, then MIC values, reported in Table 3, have been determined and compared to peptide incubated in absence of proteases. Resulting MIC values highlighted that the glycosylation of LL-III positively influence its stability by reducing the susceptibility of the parental peptide of 4 and 2 times to α-chymotrypsin and pepsin, respectively. Combining these data with the results reported in Fig. 7, it is possible to speculate that the reduced activity of peptides is due to hydrolysis of the involved amide bond and the extent/rate of hydrolysis is reduced by the presence of sugar.

**Table 3:** Comparison of minimal inhibitory concentrations (MIC, in µM) of g-LL-III and LL-III against *Escherichia coli* ATCC 25,922 after the peptides’ treatment in water (control), with α-chymotrypsin, pepsin and 50% FBS.

	MIC/µM			
	Water	α-Chymotrypsin	Pepsin	FBS
g-LL-III	3.12	6.25	6.25	3.12
LL-III	3.12	25	12.5	6.25

Moreover, we tested if the N’-terminal glycosylation of LL-III could improve peptide stability also in serum. To this end, both LL-III and g-LL-III were pre-incubated with 50% FBS for 24 h and then their residual antimicrobial activity was evaluated determining their MIC values against *Escherichia coli*. For comparison, the MIC values of untreated peptides (incubated in water) were also determined. As showed in Table 3, after 24 h of treatment in FBS, a doubled MIC value of LL-III against *E. coli* is recorded, while its N’-terminal glycosylation perfectly preserved the peptide antimicrobial activity*,* further corroborating the hypothesis that the N’-terminal protection of LL-III by the sugar moiety efficiently increases the peptide resistance against proteases.

**Figure 8:**
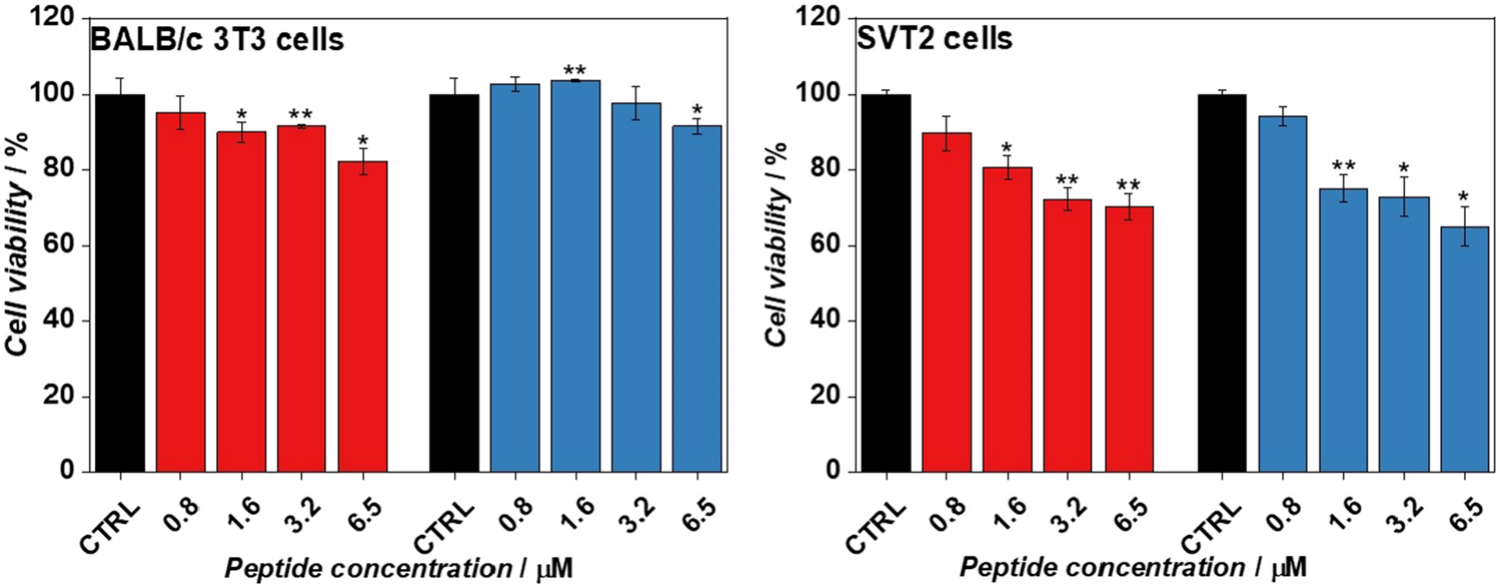
Effects of g-LL-III and LL-III on normal (BALB/C 3T3) and transformed (SVT2) murine fibroblasts. BALB/C 3T3 and SVT2 were grown in the presence of two-fold dilutions of g-LL-III and LL-III for 24 h. Cells viability values are expressed as percentage compared to control grown in medium. Three independent experiments were performed for all the experimental points, *p < 0.05 and **p < 0.01.

It was previously reported^16^ that the LL-III peptide is active not only against several bacterial strains, but also against several eukaryotic tumor cell lines. Hence, we evaluated the effect of glycosylation on the viability of both normal and tumor cells, performing the MTT assay which monitor the cells mitochondrial activity (for experimental details, please see the “Material and Methods” section). Normal and transformed murine fibroblasts, 3T3 and SVT2, were treated with increasing concentration of the two peptides. After 24 h of incubation at 37 °C and 5% CO_2_, cells mitochondrial activity was measured performing MTT assay. As reported in Fig. 8, no significant differences between the two peptides were observed for both cell lines, the 3T3 and the SVT2 cells. In conclusion, the glycosylation had not significantly influenced the activity of LL-III on eukaryotic cell lines.

## Conclusions

In this work, synthesis of the glycosylated form (NAG) of the antimicrobial peptide LL-III was carried out. Then, the interaction of g-LL-III with a model bacterial membrane composed of POPC/POPG (8/2 mol/mol) was determined to reveal similarities and differences with the unmodified peptide, whose interaction with the same model membrane has previously been reported. The overall biophysical results clearly show that the presence of NAG at the N-terminal region of the peptide does not alter its mechanism of action. The g-LL-III peptide is still able to adopt a helical conformation upon binding to the lipid bilayer, induce the formation of lipid domains within the bilayer plane, allowing the peptide to gain access to inner, cytosolic region of the cell. The biological assays show that the peptide activity on both bacterial strains and eukaryotic cell lines is unaffected by the presence of the sugar moiety. Remarkably, results on the kinetics of hydrolysis coupled with biological assays on *E. coli* after treatment with proteases or *serum* demonstrate that the glycosylation effectively increased the peptide’s resistance against the action of proteases. Overall, the reported results demonstrate that the proposed modification of the antimicrobial peptide increased the stability of the peptide to the action of proteases and provides a good starting point for further modifications (*e.g.*, attaching di- or tri-saccharides, or glycosylating other amino acid residues). Increasing the stability of the peptides to proteases without compromising their biological activity is of paramount importance for the successful application of AMPs in medicine and, possibly, in other areas of biotechnological interest, such as the food and cosmetic industry.

## Supplementary Information

Supplementary Information.

## Data Availability

All the data are available from the corresponding authors upon request.
