# Supplementary material for: The impact of N-glycosylation on the properties of the antimicrobial peptide LL-III
Source: Sci Rep. 2023 Mar 6;13:3733. doi: 10.1038/s41598-023-29984-0 (PMC9988962; doi:10.1038/s41598-023-29984-0)
Supplement: Supplementary file 1 — Supplementary Information. [file 41598_2023_29984_MOESM1_ESM.docx]

**Supporting Information**

**The impact of N-glycosylation on the properties of the antimicrobial peptide LL-III**

Attila Tortorella^1,#^, Linda Leone^1^, Angelina Lombardi^1^, Elio Pizzo^2,3^, Andrea Bosso^2^, Roland Winter^4^, Luigi Petraccone^1^, Pompea Del Vecchio^1,^* and Rosario Oliva^1,^*

^1^ Department of Chemical Sciences, University of Naples Federico II, Via Cintia 4, I-80126, Naples, Italy

^2^ Department of Biology, University of Naples Federico II, Via Cintia 4, I-80126, Naples, Italy

^3^ Centro Servizi Metrologici e Tecnologici Avanzati (CeSMA), University of Naples Federico II, Naples, 80126, Italy

^4^ Department of Chemistry and Chemical Biology, Biophysical Chemistry, TU Dortmund University, Otto-Hahn-Str. 4a, D-44227, Dortmund, Germany

^#^ Present address: Scuola Superiore Meridionale (SSM), Largo San Marcellino 10, I-80138, Naples, Italy

Corresponding authors: [pompea.delvecchio@unina.it](mailto:pompea.delvecchio@unina.it) (P.D.V.), [rosario.oliva2@unina.it](mailto:rosario.oliva2@unina.it) (R.O.)

*

*

**Figure S1** RP-HPLC chromatogram at l = 210 nm of crude LL-III peptide. The desired product is eluted at R_T_ = 25.80 min (yield = 58%).


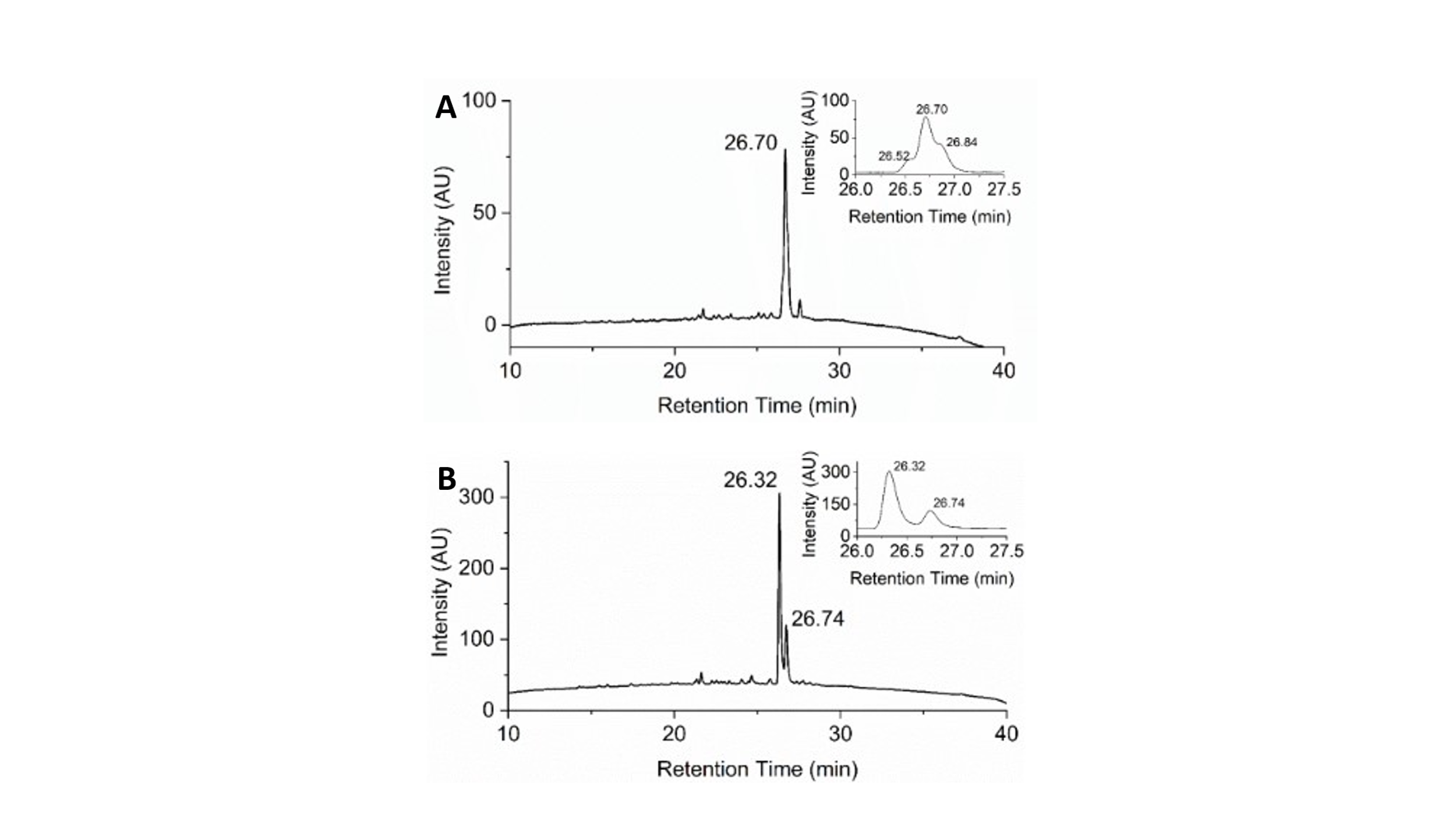


**Figure S2** RP-HPLC chromatograms at l = 210 nm of crude g-LL-III peptide before (A) and after (B) sugar deprotection. The insets show the main peaks in detail. The fully deprotected product is eluted at R_T_ = 26.32 min (yield = 63%).

*

*

**Figure S3** RP-HPLC chromatograms at l = 210 nm of pure LL-III peptide.

*

*

**Figure S4** ESI-MS spectrum of LL-III peptide. The signal at m/z = 589.4 Th corresponds to the [M+3H^+^]^3+^ ion and the signal at m/z = 883.0 Th corresponds to the [M+2H^+^]^2+^ ion. The derived experimental isotopic mass (1764.6 ± 0.6 Da) is in agreement with the theoretical value of 1764.2 Da.

*

*

**Figure S5** RP-HPLC chromatograms at l = 210 nm of pure g-LL-III peptide.

*

*

**Figure S6** ESI-MS spectrum of g-LL-III peptide. The signal at m/z = 657.1 Th corresponds to the [M+3H^+^]^3+^ ion and the signal at m/z = 985.3 Th corresponds to the [M+2H^+^]^2+^ ion. The derived experimental isotopic mass (1968.4 ± 0.2 Da) is in agreement with the theoretical value of 1968.3 Da.

**
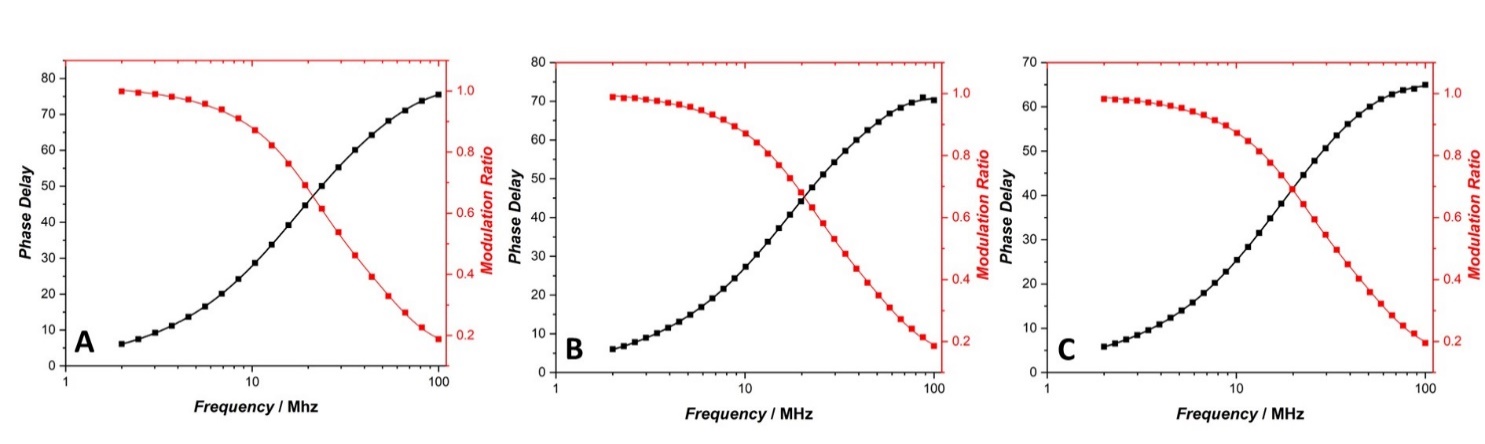
**

**Figure S7** Frequency responses (phase and modulation) of DPH (1.4 µM) embedded in POPC/POPG LUVs (50 µM), in the absence (A) and in the presence of (B) g-LL-III and (C) LL-III, at L/P = 10. The excitation was from a 370-nm LED laser. The emission was collected through a 395 nm long-pass filter. Dimethyl-POPOP was used as standard (τ = 1.45 ns). The data are best fitted with two decay times (with χ^2^ equals to 0.456, 0.901 and 1.464 for the experiments carried out in the absence of peptides, in the presence of g-LL-III and in the presence of LL-III, respectively).

**Figure S8** Kinetics of the hydrolysis of the substrate AAF-AMC performed by the enzyme α-chymotrypsin. Black line is the hydrolysis of AAF-AMC in neat buffer. The red and blue lines are instead the reaction carried in the presence of and g-LL-III, respectively. All the experiments were performed at 25°C in 10 mM phosphate buffer, pH 7.4.
